# Supplementary material for: Rhodomyrtone as a New Natural Antibiotic Isolated from Rhodomyrtus tomentosa Leaf Extract: A Clinical Application in the Management of Acne Vulgaris
Source: Antibiotics (Basel). 2021 Jan 22;10(2):108. doi: 10.3390/antibiotics10020108 (PMC7912151; doi:10.3390/antibiotics10020108)
Supplement: Supplementary file 1 [file antibiotics-10-00108-s001.pdf]

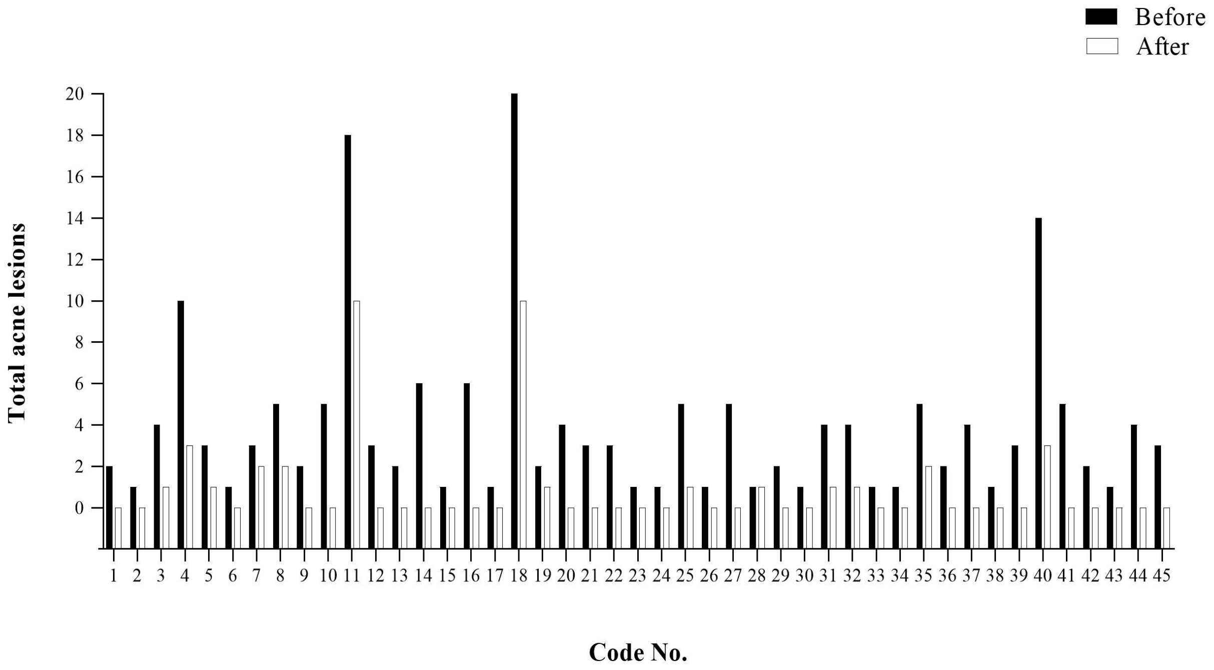

Figure S1. Quality of inflammatory acne lesions.

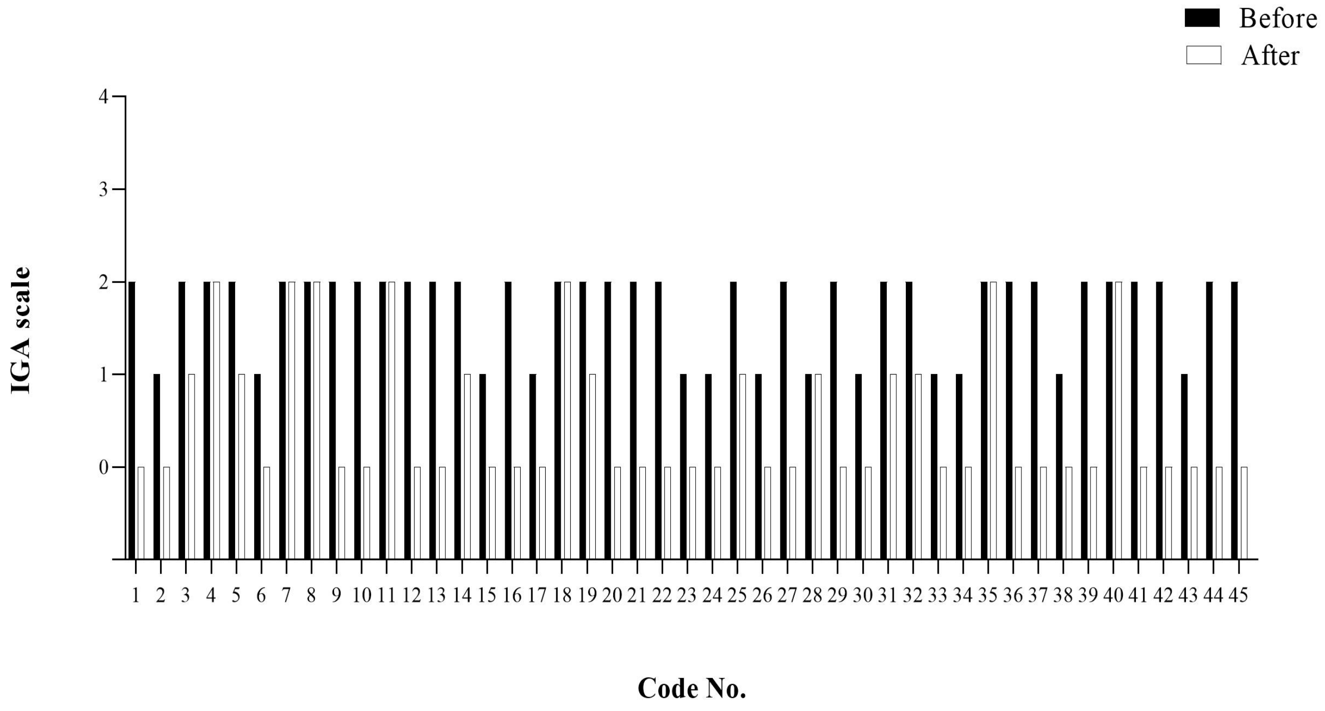

Figure S2. Quality of investigation's global assessment.
